# Silk-Cellulose Acetate Biocomposite Materials Regenerated from Ionic Liquid

**DOI:** 10.3390/polym13172911

**Published:** 2021-08-29

**Authors:** Ashley Rivera-Galletti, Christopher R. Gough, Farhan Kaleem, Michael Burch, Chris Ratcliffe, Ping Lu, David Salas-de la Cruz, Xiao Hu

**Affiliations:** 1 Department of Physics and Astronomy, Rowan University, Glassboro, NJ 08028, USA; riveragaa4@students.rowan.edu (A.R.-G.); goughc2@students.rowan.edu (C.R.G.); kaleemf7@students.rowan.edu (F.K.); burchm1@students.rowan.edu (M.B.); ratcliffc5@students.rowan.edu (C.R.); 2 Department of Chemistry and Biochemistry, Rowan University, Glassboro, NJ 08028, USA; lup@rowan.edu; 3 Department of Chemistry, Center for Computational and Integrative Biology, Rutgers University-Camden, Camden, NJ 08102, USA; david.salas@camden.rutgers.edu; 4 Department of Biomedical Engineering, Rowan University, Glassboro, NJ 08028, USA; 5 Department of Molecular and Cellular Biosciences, Rowan University, Glassboro, NJ 08028, USA

**Keywords:** silk fibroin, cellulose acetate, composite film, ionic liquid, crystalline structure

## Abstract

The novel use of ionic liquid as a solvent for biodegradable and natural organic biomaterials has increasingly sparked interest in the biomedical field. As compared to more volatile traditional solvents that rapidly degrade the protein molecular weight, the capability of polysaccharides and proteins to dissolve seamlessly in ionic liquid and form fine and tunable biomaterials after regeneration is the key interest of this study. Here, a blended system consisting of *Bombyx Mori* silk fibroin protein and a cellulose derivative, cellulose acetate (CA), in the ionic liquid 1-ethyl-3-methylimidazolium acetate (EMIMAc) was regenerated and underwent characterization to understand the structure and physical properties of the films. The change in the morphology of the biocomposites (by scanning electron microscope, SEM) and their secondary structure analysis (by Fourier-transform infrared spectroscopy, FTIR) showed that the samples underwent a wavering conformational change on a microscopic level, resulting in strong interactions and changes in their crystalline structures such as the CA crystalline and silk beta-pleated sheets once the different ratios were applied. Differential scanning calorimetry (DSC) results demonstrated that strong molecular interactions were generated between CA and silk chains, providing the blended films lower glass transitions than those of the pure silk or cellulose acetate. All films that were blended had higher thermal stability than the pure cellulose acetate sample but presented gradual changes amongst the changing of ratios, as demonstrated by thermogravimetric analysis (TGA). This study provides the basis for the comprehension of the protein-polysaccharide composites for various biomedical applications.

## 1. Introduction

The use of biodegradable polymer or biopolymer materials has been of great interest in the past decades due to the growing environmental problems posed by nonbiodegradable and petroleum-based materials. The depletion of fossil resources such as coal and natural gas and the impact of the energy crisis is becoming more severe, as seen from the ever-fluctuating price of crude oil [1]. As a response, the heightened interest in the search for renewable resources has paved the way for research into biocomposites for their biodegradability and eco-friendliness. A biocomposite is usually made of a natural biopolymer matrix and additional reinforcement element(s) to produce a composite material with enhanced properties [1]. The allure of a more inexpensive and biocompatible option as compared to the manufacturing of cost-exhaustive synthetic polymers are also a welcomed reason for their replacement [2,3]. The various applications for natural biomaterials are superior for use in the medical field as these materials are more commercially attractive and provide enhanced compatibility within the human body [4].

As a naturally occurring biopolymer, silk is a fibrous protein produced from the larvae of *Bombyx Mori* silkworms. This insect is critical to producing much of the world’s supply of silk. The primary protein fibers that silk consists of are fibroin and sericin, and its secondary structure consists almost entirely of beta-pleated sheets [5,6]. Raw silk is usually processed via a degumming procedure involving boiling the cocoons in an alkaline sodium carbonate solution to remove the water-soluble sericin layer. This degummed silk product is extremely pliable, as it can be conformed into various forms, such as gels, scaffolds, nanofibers, and composite materials. Silk is also a very tough natural fiber due to it consisting mainly of the amino acid glycine, which allows for fibers to be tightly packed together and endure less steric hindrance [4,5,7]. In the clinical setting, silk-based biomaterials are widely used due to their ease of processing, remarkable biocompatibility, adjustable degradation rates, and permeability in water and oxygen [3]. Some applications of silk include usage of anticoagulants, prosthetics, hygienic products, and arterial grafting [3,7,8]. Although silk has many applications as green or biological materials, its chemical and physical nature requires the addition of a second soft natural polymer, in this case cellulose acetate for a more structurally stable material [9].

Cellulose possesses strong inter and intra-molecular hydrogen bonding which provides the structural rigidity found in all trees and plants [10,11,12]. This biopolymer is very easy to extract, is sustainable, and has superior biocompatibility, which makes it suitable to be implemented in wound dressings. Its molecular structure contains repeating D-glucose units bonded via glycosidic linkages allowing them to fall into a tightly packed crystalline form [10,12]. Hydrophilic in nature, cellulose’s highly crystalline structure renders it insoluble in water and in many commonly used organic solvents [13]. Of the cellulose derivatives, cellulose acetate (CA) is the most studied derivative due to its chemical resistance, stability, and solubility in many organic solvents [10,11]. CA exhibits an excellent biocompatibility as a promising material suitable for immobilization of biological compounds. It has been utilized in the production of eyeglass frames, cigarette filters, and post-burn skin protectants, as well as for cardiac tissue engineering, and as a semi-permeable osmotic pump in drug-delivery systems [7,14]. Due to its low tensile moduli and solubility, CA biomaterials are enhanced when combined with other natural biopolymers, specifically silk in this case. Combining these polymers will ensure the desired products exhibit strength and flexibility; however, the proper ratios of each biomaterial must be adjusted to ensure the desired physical properties are induced [4,5].

The addition of cellulose acetate to silk means that the next palpable step is to find a solvent that will preserve the chemical characteristics of the materials. Studies have shown that ionic liquids (ILs) are adequate compounds that could successfully carry out this task [12,13,15]. Ionic liquids are charged molecules that exist as liquids at room temperatures [16]. ILs are often defined as molten salts or even liquid electrolytes with melting points below 100 °C. ILs consist of cations and anions, which differ in size and possess conformational flexibility. In such salts, crystallization is impeded by a low Gibbs free energy of crystallization, which ultimately translates into low melting points. ILs are a much safer alternative solvent to organic ones since they are thermally stable, non-flammable, and have low volatility [12]. It is also known that ionic liquids can stabilize proteins and maintain their molecular weights. Specifically, ionic liquid mediated hydrogen bonds prevent breakdown of protein structure, even when surpassing extreme temperature thresholds [7,14]. These novel properties of ILs lead to its use as a solvent for several different biomaterial systems based on proteins, polysaccharides, and their composites [12,17,18,19,20,21,22,23,24,25,26,27,28,29,30,31,32,33,34,35,36,37].

Among the considerable number of varying ILs explored today, only a minority can dissolve cellulose effectively [38]. Specifically, a small number of suitable anions are possible for the dissolution of both protein polymers and cellulose-based polymers. In this study, the ionic liquid 1-ethyl-3-methylimidazolium acetate (EMIMAc) was used to dissolve silk and cellulose-acetate, where the acetate anion is known to act as a catalyst in the ring opening reaction of cellulose [38]. Thin films consisting of silk and cellulose acetate ratios were made and characterized using the Fourier-transform infrared spectroscopy (FTIR). Thermal properties were analyzed using differential scanning calorimetry (DSC) and thermal gravimetric analysis (TGA). A high-resolution image of surface topography and composition of these films, on a microscopic level, was produced via the implementation of a scanning electron microscope (SEM). Results of these different experimental analyses will produce viable information on the ability to deploy silk cellulose-acetate biomaterials into the fields of biomedical and sustainable material engineering.

## 2. Materials and Methods

### 2.1. Raw Materials

*Bombyx mori* silk cocoons were purchased from Treenway Silks (Lakewood, CO, USA). The silk cocoons were boiled in 0.02 M NaHCO_3_ obtained from Sigma Aldrich USA (CAS#: 144-55-8) for 15 min, then washed three times in deionized water baths in order to remove sericin proteins and extract the silk fibroin. Following this, the silk fibers were dried in a fume hood for 48 h. Cellulose acetate powder (CAS#: 9004-35-7) and 1-ethyl-3-methylimidazolium acetate (EMIMAc) (CAS#: 143314-17-4) were purchased from Sigma Aldrich Co., LTD (St. Louis, MO, USA). Methanol was purchased from Sigma Aldrich USA (CAS#: 67-56-1). Prior to being used as a solvent, EMIMAc was placed in a vacuum oven at 60 °C for 24 h to fully remove any residual water moisture. All the substances that were used for the chemical analysis were analytical grade.

### 2.2. Film Preparation

**Figure 1:**
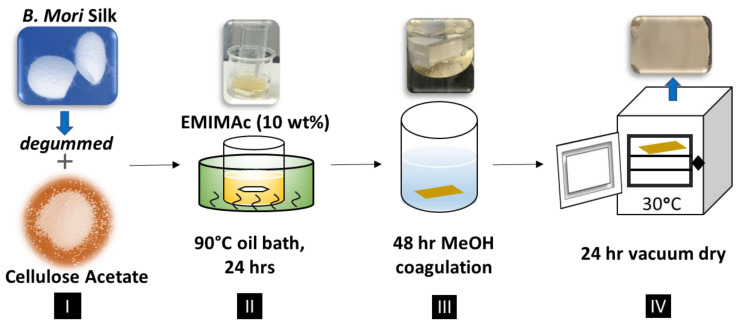
Procedure for the fabrication of silk-cellulose acetate films using EMIMAc as solvent.

For preparing composite film samples, a total of 3 g of the blended ionic liquid EMIMAc with solids (raw materials) was implemented, in which the ratios required 90% ionic liquid to 10% of solid materials. Once the ratio of ionic liquid to solids had been determined, the selected solid materials would be submerged and dissolved into the ionic liquid. A total of seven weight ratios were selected as the solid materials: 100% Cellulose Acetate (CA100), 90% Cellulose Acetate–10% Silk (CA90S10), 75% Cellulose Acetate–25% Silk (CA75S25), 50% Cellulose Acetate–50% Silk (CA50S50), 25% Cellulose Acetate–75% Silk (CA25S75), 10% Cellulose Acetate–90% Silk (CA10S90), and 100% Silk (Silk100). The ionic liquid was then fully submerged into a silicon oil bath, ranging from 70–80 degrees Celsius, prior to adding the solids. This was then followed-up with a 24-h mixing period [1,4]. This process is shown below in Figure 1.

The solids were added in the order of proteins first, followed by carbohydrates. Once the 24-h heating period was completed, all the ionic liquids were removed from the biocomposite films via a coagulation bath. The coagulation bath used in this study was methanol. For the water coagulation baths, the samples had dissolved partially during the process and were not able to be dried to form composite films. Thus, only samples washed in methanol were able to form solid biomaterials and investigated in this study. The samples were continually washed inside of the methanol coagulation baths for 48 h. After 48 h, the film samples were removed from the methanol coagulation baths and placed in a vacuum for 24 h to dry the films.

### 2.3. Fourier Transform Infrared Spectroscopy (FTIR)

FTIR analysis of the silk and cellulose acetate films was conducted by using a Bruker Tensor 27 Fourier Transform Infrared Spectrometer (Billerica, MA, USA). The FTIR spectrometer had an addition of a triglycine sulfate detector and a multiple reflection, horizontal MIRacle ATR attachment (using a Ge crystal, from Pike Tech. (Madison, WI, USA)). For each sample measurement, a total of 64 background scans and 64 sample scans were taken from the 4000 cm^−1^ to 400 cm^−1^ range at a resolution 2 cm^−1^. To ensure a homogeneous distribution in the films, samples were taken from multiple spots and sides in triplicate at room temperature (~20 °C). The Ge crystal was cleaned with ethanol and dried between samples. To process the results, spectra from each sample were analyzed using the OPUS software [16,39].

### 2.4. Differential Scanning Calorimetry (DSC)

Roughly 6 mg of thin film samples were enclosed in an aluminum pan and pressed closed to prepare for DSC analysis. A Q100 DSC (TA Instruments, New Castle, DE, USA) equipped with refrigerated cooling system was used, with 50 mL/min of nitrogen purge gas flowed through the sample chamber. Prior to use, the instrument was calibrated with an indium crystal for heat flow and temperature, while aluminum and sapphire standards calibrated the heat capacity. Temperature-modulated differential scanning calorimetry (TMDSC) measurements were performed at a heating rate of at 2 °C/min with a modulation period of 60 s and temperature amplitude of 0.318 K, from −40 °C to 400 °C. The Lissajous figures of modulated heat flow vs. modulated temperature were also plotted to check the establishment of steady state. This gave data regarding the heat flow and reversing heat capacity versus the temperature. This test was then run with the seven different samples that were produced [5,39]. In order to confirm the reliability of the experiment, the samples were tested three times for each condition.

### 2.5. Thermal Gravimetric Analysis (TGA)

Thermogravimetric analysis (TGA) of composite films was investigated with a TA Instruments Q600 SDT instrument (Wilmington, DE, USA). The TGA had a precision balance with a small ceramic pan inside of the furnace. The furnace temperature was controlled to increase the temperature from 25–800 °C at a rate of 10 °C/min. Nitrogen purge gas was used at a rate of 50 mL/min. The mass of the samples was measured over time with regards to changing temperatures in order to measure the thermal stability of the samples [39,40]. In order to confirm the reliability of the experiment, each type of sample was tested three times.

### 2.6. Scanning Electron Microscopy (SEM)

A FEI VolumeScope™ SEM (Hillsboro, OR, USA) was used in assessing the morphology of the bio-composites. The FEI SEM implements four different beam currents that are directed towards the sample of interest. This allows the SEM to show the morphology of the blended films with details on the microscopic level. The samples were placed on SEM holders and held into place with circular conducting tape. They were then coated by a thin layer of gold in the Denton Vacuum Desk sputtering machine for a spell of 10~90 s. The samples were then placed into the SEM and prepared for imaging at room temperature under high vacuum. Experiments were conducted with an accelerating voltage ranging between 10 and 20 kV.

## 3. Results and Discussion

### 3.1. Morphological Analysis

The physical appearance of the bulk silk-cellulose acetate bio-composite films displayed a common trend. The pure silk sample was smooth and unyielding while the pure cellulose acetate sample was thin and flexible, confirming cellulose acetates more pliable properties. The physical properties of composite materials can be adjusted depending on the amount of CA added to the silk film. The topography of the silk dominated composite films (CA10S90, CA25S75) displayed ridges and grooves while the cellulose acetate dominated films (CA90S10, CA75S25) displayed a gradual increase in ridges and grooves when mixed with silk. The silk dominated films were brittle yet also strong and with increasing cellulose acetate showed more flexibility.

**Figure 2:**
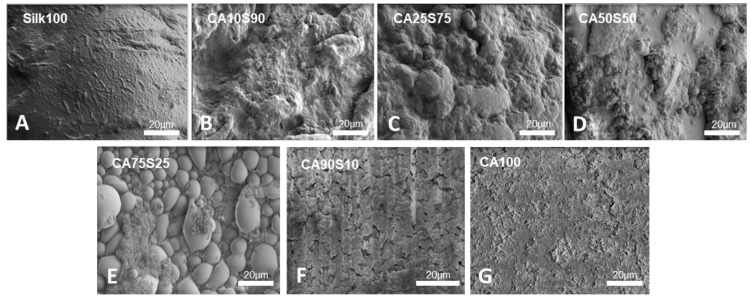
Scanning electron microscope (SEM) images of methanol treated composite samples of silk and cellulose acetate and their respective pure samples. (**A**) Silk 100, (**B**) CA10S90, (**C**) CA25S75, (**D**) CA50S50, (**E**) CA75S25, (**F**) CA90S10, (**G**) CA100.

To further investigate the surface morphology of composite films at microscale, the methanol-coagulated samples were analyzed by SEM, as shown in Figure 2. For the composite films, significant surface morphology change was observed as compared to the pure samples shown in Figure 2. The pure cellulose acetate sample (CA100) had a homogeneous surface with smooth and continuous ridging. The pure silk sample (Silk100) also displayed a homogenous and smoother surface. In Figure 2, all composite films become rougher generally on the microscopic scale. With just 10% of cellulose acetate added, the 90% silk sample (CA10S90) has a slightly porous and rough topography. The 75% silk (CA25S75) showed a similar appearance and the 50% sample (CA50S50) had intermittent smooth sections formed amongst the roughness. As cellulose acetate began to dominate in the sample (CA75S25), the topography showed a smoother surfaced cobble stone or closely forming bubbled appearance. The 90% CA dominated film (CA90S10) showed a similar topography to its 100% CA (CA100) counterpart with a more continuous and smoother appearance with slight cracking. The trend seems to indicate that with just small increments of CA added, the surface can undergo a drastic change in topography from smooth to rough and porous, leading to potential in applications regarding organic filters and cell culture growth studies.

### 3.2. Structural Analysis

**Figure 3:**
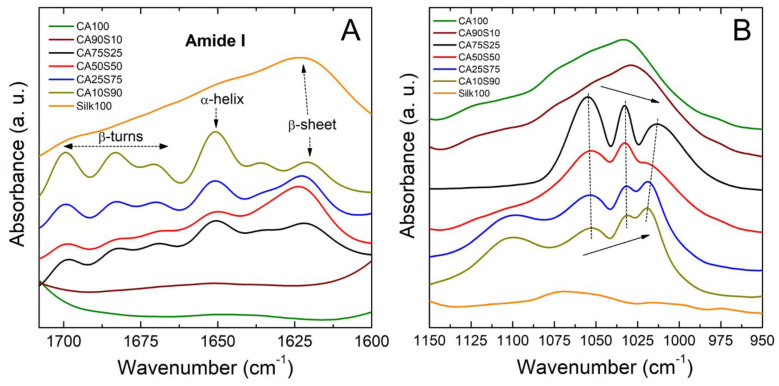
FTIR spectra for silk-cellulose acetate films made using a methanol bath. (**A**) The region containing the amide I (1700–1600 cm^−1^) peaks of silk proteins. (**B**) The region depicted is related to the structure of cellulose in the 1150 cm^−1^ to 950 cm^−1^ range.

Structural changes in silk-cellulose acetate films after methanol coagulation were confirmed by FTIR and shown in Figure 3. The IR spectral region within 1700–1600 cm^−1^ is assigned to the peptide backbone of amide I (1700–1600 cm^−1^) and amide II (1600–1500 cm^−1^) absorption (Figure 3A), which have been commonly used for the analysis of different secondary structures of silk fibroin proteins. The peaks at 1630–1610 cm^−1^ (amide I) and 1520–1510 cm^−1^ (amide II) are characteristic of silk II structure (dominated by beta-sheets) [41,42]. Pure silk film (CA0S100) is dominated by beta-sheet crystalline structure (around 1620 cm^−1^). The addition of cellulose acetate increased the alpha-helical structure (around 1650 cm^−1^) from beta-sheet formation, probably due to the hydrogen bonding of acetate with the protein chains of silk as seen in Figure 3A, while pure cellulose acetate (CA100S0) did not show strong absorbance in this region.

Further analysis on the structural composition of silk-cellulose acetate films was characterized within the region of 1050–1000 cm^−1^, as shown in Figure 3B. This region is often chosen as the reference characteristic peak due to its stable position and intensity even during CA acetylation [43]. Pure cellulose acetate films show a dominant peak at 1031 cm^−1^, which is attributed to the C-O-C stretching vibration in the backbone of anhydroglucose units [43]. The addition of silk proteins splits the peak into three peaks around 1054, 1032, and 1020 cm^−1^. Meanwhile, with the increase of silk content, the intensity of the left peak at 1054 cm^−1^ gradually weakened, and the intensity of the right peak around 1020 cm^−1^ gradually increased. The position of the right peak also shifts to the right from 1020 to 1013 cm^−1^ as the cellulose acetate content increases in the films. These spectral changes may be another indicator of the silk-CA molecular interactions in the composite films. Although these hydrogen bond interactions mainly occur on the side chain groups, the C-O-C stretching vibration in the CA backbone is also affected.

### 3.3. Thermal Analysis

**Figure 4:**
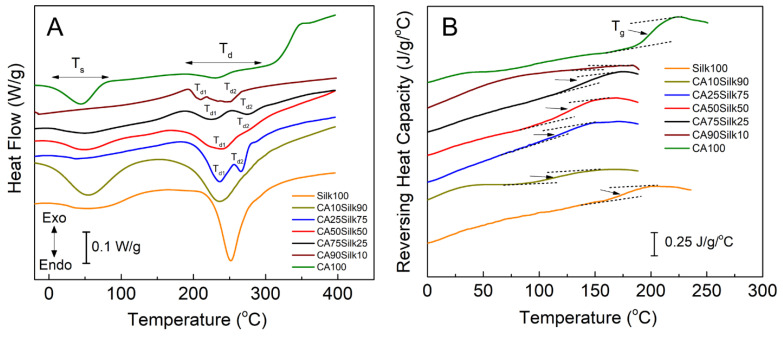
DSC scans of (**A**) heat flow and (**B**) reversing heat capacity for methanol washed silk-cellulose acetate composite films.

Temperature-modulated DSC (TMDSC) was performed to further understand thermal properties of the silk-cellulose acetate composite films, and its results are shown in Figure 4 and Table 1. When observing Figure 4A, the solvent release temperature, T_s_, refers to the release temperature where most of the bound water/solvent molecules evaporated through the heating process. The second label, T_d_, refers to the degradation temperature, at which the sample begins to thermally degrade because of the heating. It is notable that at the point of degradation (T_d_) of the 100% silk sample, there is only one defined peak, but when the cellulose acetate is added to the silk film, that single degradation peak turns into two peaks (T_d1_, T_d2_). This indicates that each of the two individual samples still maintained their unique chemical properties of polymer backbones through the mixing in EMIMAc ionic liquid. However, the positions of the two degradation peaks (T_d1_, T_d2_) are different from that of the individual pure silk or CA materials (Table 1), suggestion that the strong molecular interactions (such as hydrogen bonds) exist between the silk and CA side chain groups. Figure 4B shows the reversing heat capacity scans of different silk-CA samples, which clearly demonstrated the glass transitions of the composites. Further analysis of these glass transition regions shows that with the increase of CA content, the glass transition temperature (T_g_) of composites increases slightly, from 113.6 °C for CA10S90 film to 200.1 °C for pure cellulose acetate film (CA100). However, the T_g_ of the pure silk films (Silk100) did not follow this trend, with a value of 178.5 °C, which is like the T_g_ values found in silk films regenerated from water or organic solvents [41,42]. As previously mentioned in the FTIR analysis, the pure silk films contained the largest beta-sheet crystallinity, making them overall the most thermally stable of all composite films.

### 3.4. Thermal Stability

**Figure 5:**
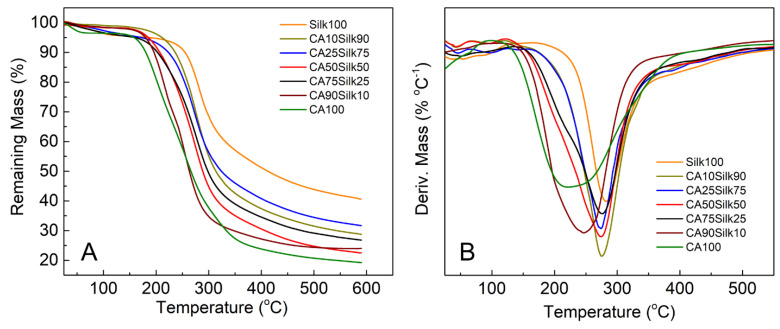
Thermogravimetric analysis of silk-cellulose acetate films: (**A**) remaining mass percent of samples with the increasing temperature; (**B**) the first derivative of the remaining mass curves.

Additional analyses of the thermal properties of the silk-cellulose acetate films were conducted by TGA as shown in Figure 5. Table 1 also summarized several typical TGA parameters including onset temperature of decomposition, bound solvent content, degradation middle temperature (T_dm_), and remaining mass at 400 °C. Each sample followed the same decomposition trend as the DSC results where the pure silk sample maintained the best thermal stability after undergoing higher temperatures as compared to the blended films. When silk is dominant, the blend samples have a greater onset decomposition temperature, and the degradation middle temperature (T_dm_) is also higher. Once the concentration of cellulose acetate is equal to or greater than that of silk, the remaining mass at 400 °C is much lower, indicating that cellulose acetate component greatly reduces the thermal stability of the blended samples at high temperatures.

The pure silk and pure cellulose samples tended to be on the opposite ends of the spectrum where silk is most stable and pure cellulose acetate was weakest. This could be due to the hydrophobic nature of cellulose acetate. The combination of cellulose acetate with a small amount of silk leads to a significant shift in the degradation middle temperature (T_dm_) peak to a higher degree (as seen in Figure 5B). This suggests more thermal stability can be easily achieved with just 10~25% of *B. Mori* silk added into the composites.

### 3.5. Mechanism

**Figure 6:**
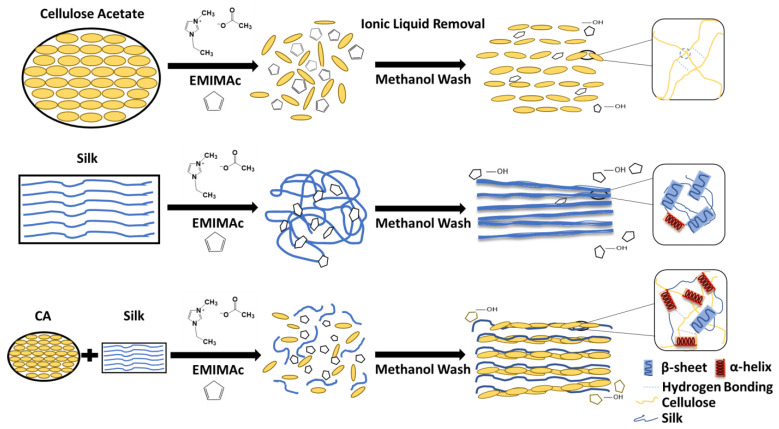
Proposed structural mechanism for the mixing of silk and cellulose acetate using EMIMAc as the solvent.

Based on the results outlined above, the proposed mechanism for silk-cellulose acetate biocomposites can be seen in Figure 6. Immediately after dissolution in ionic liquid, cellulose acetate takes on a more disordered structure and the molecular chains are expanded, ready to interact with silk molecules. Meanwhile silk fibroin natural fibers are also dissolved in the selected ionic liquid (EMIMAc), and the beta sheet crystals are disassembled into soluble structures such as random coils, alpha helix, and beta turns. After washing by methanol, both pure cellulose acetate and silk materials can regain their ordered molecular structure, confirmed by FTIR (e.g., silk molecules will form dominant insoluble beta-sheet crystals in the structure). With the increase of the cellulose acetate content in the composite material, the possibility of the formation of hydrogen bonds between the CA chain and the silk chain increases, so more α-helical structures are formed, which inhibits the formation of β-sheet crystals in the biocomposites. According to the results of FTIR and thermal analysis, during this process, silk and CA molecules were successfully mixed without immiscible phase separation, which significantly improved the stability of the composite structure.

## 4. Conclusions

Using EMIMAc as an ionic liquid solvent, *Bombyx mori* silk and cellulose acetate are both able to be dissolved into the same solvent to form composite biomaterials with the benefits of each individual biopolymer. Various ratios of silk to cellulose acetate were able to be fabricated into composites, with tunable structures confirmed by characteristic FTIR peaks of both cellulose acetate and silk in the blended samples. The effects of methanol as a coagulation agent were observed in the structural analysis of the composites. Both FTIR and SEM reveal how the crystallinity and morphology of the composite varies with ratio of silk to cellulose. These effects also affect the thermal properties of the composites, where silk-heavier samples have a higher degradation temperature due to the higher beta-sheet content of silk. Of special note related to the use of ionic liquids, the composite samples exhibit single glass transition temperatures and changing thermal degradation peaks as seen in the DSC analysis, indicating strong interactions between silk and cellulose acetate molecules. This study proves the potential of EMIMAc as a solvent for *Bombyx mori* silk and cellulose acetate, and how its regeneration in methanol results in practical biocompatible films for various applications.

## Figures and Tables

**Table 1:** Thermal analysis data of different silk-cellulose acetate films produced from EMMIAc *.

Sample	T_g_/°C	Solvent Release T_s_/°C	Degradation Temperature T_d_/°C	Onset Temperature of Decomposition/°C	Bound Solvent Content/%	Degradation Middle Temperature T_dm_/°C	Remaining Mass at 400 °C/%
**Silk100**	178.5	54	252	180	5.5	283	51.2
**CA10S90**	113.6	53	237	161	1.1	275	37.4
**CA25S75**	117.2	41	236/266	160	4.3	274	40.7
**CA50S50**	123.5	49	239/276	138	1.8	274	30.6
**CA75S25**	141.7	50	225/275	141	4.8	273	34.6
**CA90S10**	154.4	N/A	211/250	132	1.9	247	27.2
**CA100**	200.1	46	230	123	3.6	223	23.8

* All numbers have an error bar within ±5%. The first three columns (T_g_, T_s_, and T_d_) were determined by TMDSC analysis, the rest were determined by TG analysis. T_g_, T_s_, and T_d_ represent the glass transition temperature, bound solvent release peak temperature, and degradation peak temperature of different silk-CA films, respectively.

## Data Availability

The data presented in this study are available on request from the corresponding author.
